# Comprehensive characterization of tobacco-induced changes in enamel surface topography

**DOI:** 10.1016/j.jobcr.2024.12.007

**Published:** 2024-12-24

**Authors:** Tamanna Kaur, Ramya Ramadoss, Nitya Krishnasamy, Sandhya Sundar, Suganya Panneer Selvam, Hema Shree K

**Affiliations:** a Department of Oral Biology, Saveetha Dental College and Hopsitals, Saveetha Institute of Medical and Technical Sciences, Saveetha University, Chennai, 600077, India; b Department of Oral Biology and Oral Pathology, Saveetha Dental College and Hopsitals, Saveetha Institute of Medical and Technical Sciences, Saveetha University, Chennai, 600077, India

**Keywords:** Enamel translucency, Surface roughness, Spectrophotometry, Atomic force microscopy (AFM), Stylus profilometry, Tobacco staining, Optical properties

## Abstract

**Introduction:**

Enamel translucency, essential for the aesthetic appeal of teeth, is primarily determined by its thickness, quality, and refractive index. Several factors, including age, genetics, diet, oral hygiene practices, fluoride exposure, and acidic challenges, can influence enamel translucency. Tobacco use, in particular, leads to significant alterations in enamel appearance by penetrating its micropores, causing yellowing and browning. Prolonged exposure to tobacco results in demineralization, increasing enamel porosity and reducing its translucency. Over time, this exposure leads to permanent discoloration and structural degradation, rendering teeth dull and opaque. To assess these changes, various methods such as visual examinations, digital photography, spectrophotometry, stylus profilometry, and atomic force microscopy (AFM) are utilized.

**Materials and methods:**

This study analyzed human enamel samples, including one unstained sample (Grade 0) and three tobacco-stained samples (Grades 1–3) according to Modified Lobene Stain Index (MLSI), to investigate the impact of tobacco exposure on enamel structure. The samples were thoroughly cleaned and dried to ensure accurate results. High-resolution AFM imaging was employed to assess surface roughness, porosity, and microstructural changes induced by tobacco staining. A stylus profilometer was used to trace the surface topography, providing detailed measurements of surface irregularities. Additionally, a spectrophotometer was utilized to evaluate the optical properties of the enamel, focusing on changes in translucency and light absorption due to tobacco exposure.

**Results:**

AFM analysis revealed a clear progression of enamel surface roughness from Grade 0 to Grade 3. The Sa and Sq values increased significantly with the severity of tobacco staining, indicating greater surface degradation. The stylus profilometer data corroborated these findings, with rising Ra values as the degree of staining intensified, highlighting the topographical alterations caused by tobacco exposure. Spectrophotometric analysis further demonstrated a decrease in enamel reflectance and an increase in light absorption from mild to severe staining, underscoring the detrimental optical effects of tobacco on enamel.

**Conclusion:**

The combined use of AFM, stylus profilometry, and spectrophotometry provided a comprehensive assessment of the impact of tobacco staining on enamel translucency and surface roughness. The findings show that as staining severity increases, enamel translucency diminishes, and surface roughness worsens. These alterations not only affect the aesthetic appearance of teeth but also have potential implications for enamel functionality and long-term oral health.

## 1 Introduction

Tobacco use significantly impacts oral health, particularly through the discoloration and staining of teeth. Enamel, the tooth's outermost layer, is naturally translucent, allowing light to pass through and reflect off the underlying dentin, giving teeth their distinct colour and sheen.^1^ However, prolonged tobacco use disrupts this translucency, leading to structural and cosmetic changes. Substances such as tar and nicotine from tobacco smoke adhere to the enamel, seeping into micropores and creating intrinsic stains that are challenging to remove.^2^^,^^3^ Over time, the acidic components in tobacco also contribute to demineralization, increased enamel porosity, roughness, and a decrease in translucency, leading to permanent discoloration and weakening of the enamel's surface. Enamel, with its refractive index of about 1.62, interacts with light in complex ways, and tobacco use alters these interactions, further impacting its translucent qualities.^4^^,^^5^

Enamel translucency is influenced by various factors, including genetic predispositions, enamel thickness, and age-related wear.^6^^,^^7^ Pigmented foods, poor dental hygiene, and conditions such as dental fluorosis also contribute to enamel discoloration by altering its optical properties.^8^, ^9^, ^10^ As enamel thins over time or due to chemical exposure, more dentin is exposed, making teeth appear darker or more yellow.^11^ Dental treatments and restorative materials attempt to replicate enamel's natural translucency, but variations in optical qualities often result in differences in appearance, especially when factors like enamel thickness and refractive index are affected by tobacco use.^12^ Scanning Electron Microscopy (SEM) and Atomic Force Microscopy (AFM) are among the tools used to assess changes in enamel translucency and roughness caused by tobacco.^13^^,^^14^

Current research on enamel translucency in tobacco users is limited, primarily focusing on staining rather than the comprehensive optical changes tobacco causes.^15^ While studies show that tobacco smoke increases enamel roughness and alters its chemical makeup, there is a lack of detailed chemical analyses of specific tobacco components responsible for these changes. Furthermore, few studies have explored the microscopic structural alterations in enamel, with a notable absence of long-term longitudinal data.^16^

The objective of this study was to systematically investigate the impact of tobacco staining on enamel surface topography and translucency using advanced analytical techniques. By examining human enamel samples classified into unstained (Grade 0) and varying levels of tobacco staining (Grades 1–3), the study aimed to quantify changes in surface roughness, porosity, and optical properties. Advanced tools such as atomic force microscopy (AFM), stylus profilometry, and spectrophotometry were utilized to measure these parameters, providing a detailed characterization of the structural and aesthetic degradation caused by tobacco exposure. This comprehensive approach sought to elucidate the relationship between staining severity and enamel alterations, offering insights into the mechanisms by which tobacco affects enamel quality and appearance.

## 2 Materials and methods

### 2.1 Sample collection and classification

Enamel samples were collected from human teeth to investigate tobacco-induced alterations in surface topography. The sample set consisted of four teeth: one unaffected (Grade 0) and three tobacco-stained teeth, classified into Grades 1, 2, and 3, based on the severity of staining.

Teeth staining can be graded based on the severity and extent of discoloration (According to Lobene, RR).^16^ **Grade 0** represents no visible discoloration or staining on the tooth surface. **Grade 1 (Mild Staining)** is characterized by light brown or yellow discoloration, typically limited to localized spots or minor areas, covering less than 25 % of the tooth surface. **Grade 2 (Moderate Staining)** involves brownish or dark yellow discoloration noticeable on multiple parts of the tooth, covering 25–50 % of the surface. **Grade 3 (Severe Staining)** is marked by intense dark brown or black discoloration, prominently affecting over 50 % of the tooth surface, often impacting both aesthetics and texture.These teeth were sourced from the tooth repository at Saveetha Dental College & Hospitals. This classification provided a comparative framework for evaluating the impact of tobacco exposure on enamel structure and its physical properties.

### 2.2 Sample preparation

The enamel samples were thoroughly cleaned and dried before analysis to ensure the removal of any contaminants that could affect the results. This step was essential for achieving accurate, uncontaminated measurements of enamel surface characteristics.

### 2.3 Atomic force microscopy (AFM)

Surface characterization was performed using an Atomic Force Microscope (AFM) (Anton Paar) to obtain high-resolution images of the enamel surface. The samples were positioned under the AFM probe to measure surface roughness, porosity, and microstructural changes. The AFM data provided insights into the enamel's structural integrity and the extent of damage caused by tobacco exposure.

### 2.4 Stylus profilometry

A stylus profilometer (Mitutoyo) was used to meticulously trace the enamel surface to quantify surface roughness and the depth of any pits or grooves resulting from tobacco staining. The profilometry data were compared between the stained and unstained samples to assess the physical changes in enamel topography induced by tobacco use.

### 2.5 Spectrophotometry

**Fig. 1:**
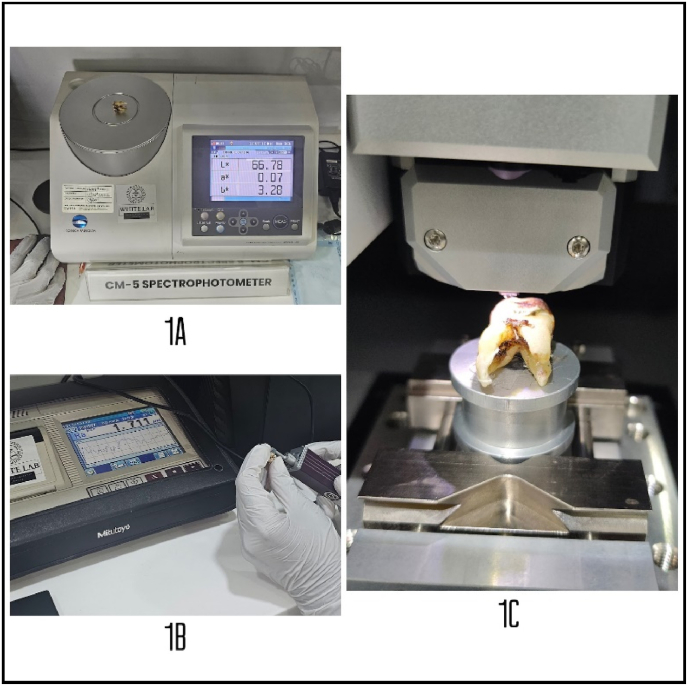
Fig. 1A: Spectrophotometer, Fig. 1B: Stylus profilometer, Fig. 1C: AFM

To evaluate alterations in the enamel's optical properties, a CM-5 spectrophotometer (ZM3.8 SD Mechatronik) was utilized. Light was transmitted through the enamel surface, and both transmission and reflection spectra were recorded. This analysis was used to measure the enamel's translucency, which can be affected by tobacco-related staining and structural modifications (Fig. 1A-C). The spectrophotometry data were compared across samples to identify variations attributable to tobacco exposure.

## 3 Results

### 3.1 Atomic force microscopy

This study employs Atomic Force Microscopy (AFM) to examine the impact of tobacco use on enamel surface topography in human teeth. The analysis compares an unaffected tooth (Grade 0) with three teeth exhibiting varying levels of tobacco staining (Grades 1–3). AFM imaging reveals that the Grade 0 sample, representing unstained enamel, displays low values for both surface roughness parameters—average roughness (Sa) and root mean square roughness (Sq)—indicating a smooth and intact enamel surface.

**Fig. 2:**
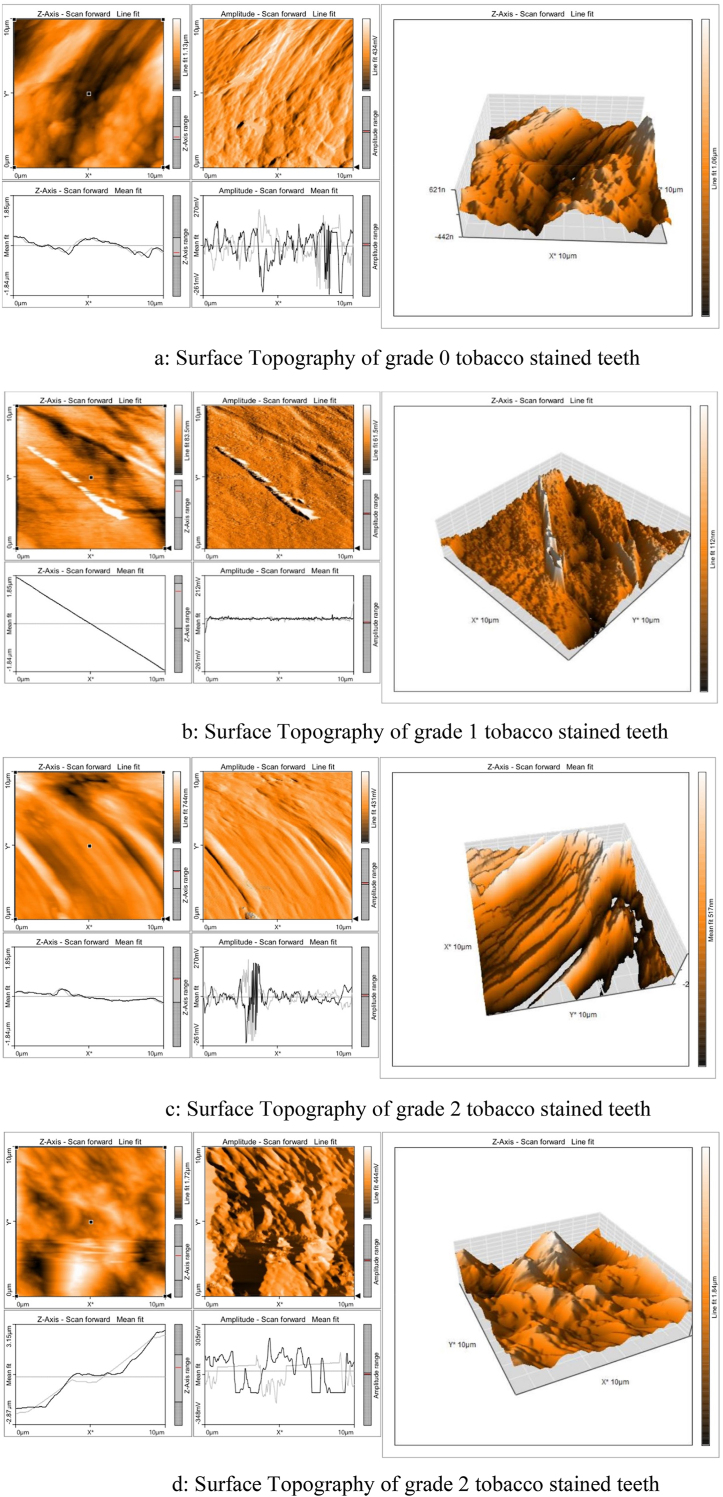
(a) Surface Topography of grade 0 tobacco stained teeth. (b) Surface Topography of grade 1 tobacco stained teeth. (c) Surface Topography of grade 2 tobacco stained teeth. (d) Surface Topography of grade 2 tobacco stained teeth.

**Graph 1a:**
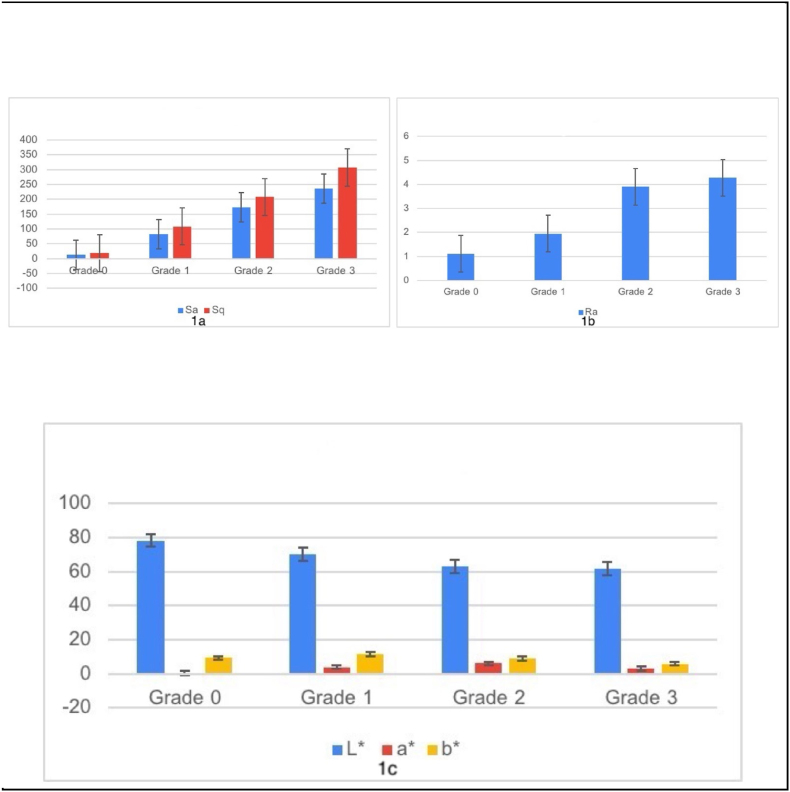
roughness values by AFM. Graph 1b: roughness values by Stylus profilometer Graph 1c: color values using spectrophotometry. (For interpretation of the references to color in this figure legend, the reader is referred to the Web version of this article.)

In Grade 1, an increase in Sa and Sq values signifies the onset of mild surface roughness, as tobacco stains begin to cause minor irregularities detectable through AFM. Grade 2 shows a further increase in these values, reflecting moderate roughness characterized by more pronounced peaks and valleys due to the accumulation of staining agents. By Grade 3, the Sa and Sq values reach their highest levels, indicating severe surface degradation with deep grooves and significant topographical changes resulting from heavy tobacco staining. Quantitative analysis of surface roughness parameters shows a marked increase from 12.555 (Sa) and 17.873 (Sq) in Grade 0 to 236.65 (Sa) and 307.5 (Sq) in Grade 3 (Fig. 2 a-d) underscoring the substantial degradation of enamel quality due to tobacco exposure. This significant rise in surface roughness highlights the detrimental effects of tobacco smoking on enamel integrity (Graph 1a-c , Table 1).

**Table 1:** Roughness values by AFM,roughness values by Stylus profilometer, colour values using Spectrophotometry.

Samples	Sa(Surface Roughness Average)	Sq(Root Mean Square Roughness)	Ra(Arithmetic Average Roughness)	L∗(Lightness)	a∗(Green - Red Component)	b∗(Blue - Yellow Component)
Grade 0	12.555	17.873	1.117	78.23	0.54	9.38
Grade 1	81.72	108.56	1.949	70.17	4.02	11.56
Grade 2	173.48	207.83	3.907	62.9	6.08	9.01
Grade 3	236.65	307.5	4.279	61.64	3.16	6.01

### 3.2 Stylus profilometer analysis

The stylus profilometer analysis revealed a progressive increase in surface roughness (Ra) values across enamel samples with varying levels of tobacco staining. Grade 0, representing unaffected enamel, exhibited the lowest Ra value of 1.117, indicating a smooth, intact surface. In contrast, Grade 1 showed a higher Ra value of 1.949, reflecting the early stages of tobacco-induced surface changes, where mild staining begins to affect enamel smoothness. As the severity of staining increased, Grade 2 displayed a significant rise in Ra value to 3.907, indicating more pronounced topographical alterations likely due to tobacco residue accumulation. The highest Ra value of 4.279 was recorded in Grade 3, corresponding to severe roughening and surface degradation from prolonged tobacco exposure. These findings underscore a direct correlation between the degree of tobacco staining and enamel roughness, with increasing Ra values signifying greater damage to the enamel's smoothness and structural integrity (Graph 1b).

### 3.3 Spectrophotometry

The spectrophotometer results (graph 1c) provided valuable insights into the optical changes in enamel surfaces due to tobacco-induced staining. Each grade of staining, from Grade 0 to Grade 3, showed distinct variations in the enamel's reflectance and absorption properties, which directly correlated with the extent of discoloration. The L∗ value measures lightness, with 0 being black and 100 being white. The a∗ value indicates the position between green and red, where negative values denote green and positive values denote red. The b∗ value represents the position between blue and yellow, with negative values indicating blue and positive values indicating yellow. Grade 0 (Unaffected Tooth) set the baseline, showing high reflectance and minimal absorption, indicating a bright, clean enamel surface with no discoloration, thus confirming the optimal optical properties of an unaffected tooth. Transitioning to Grade 1 (Mild Staining), there was a slight decrease in reflectance and increased absorption, particularly in the yellow-brown spectrum, causing the enamel to lose some brightness and take on a faint yellowish tint. Grade 2 (Moderate Staining) displayed a considerable drop in reflectance and increased absorption in the brown to dark brown regions, resulting in more pronounced yellow-brown discoloration and reduced translucency, making the enamel appear visibly darker. Finally, Grade 3 (Severe Staining) revealed substantial reductions in reflectance and high absorption, especially in the dark brown to near-black spectrum, causing the enamel to appear dark brown to almost black, indicating extensive surface and subsurface changes and significantly diminished light reflection.

## 4 Discussion

Tobacco use remains one of the most significant threats to global public health, with about one-third of the world's adult population using tobacco in some form. Of these individuals, approximately half will die prematurely due to nicotine addiction. The World Health Organization (WHO) estimated that 4.9 million people died from nicotine-related causes in 2000 alone. Tobacco use is a primary contributor to numerous chronic conditions, including various oral diseases. Epidemiological studies in India have demonstrated that up to 80 % of oral cancer patients are tobacco users.^17^ The effects of cigarette smoking on the oral cavity extend beyond oral cancer, as it contributes to tooth discoloration and significantly increases the risk of periodontal disease, a major cause of tooth loss. Human tooth enamel, primarily composed of carbonated hydroxyapatite (c-HAP), is vulnerable to the harmful effects of tobacco, which leads to progressive structural deterioration.^14^^,^^18^

Research shows that cigarette smoke exposure increases enamel wear, with formulations like fluoride-containing toothpaste offering some degree of protection. Studies using atomic force microscopy (AFM) have demonstrated that smokers exhibit rougher enamel surfaces compared to non-smokers.^19^ AFM quantitative analysis revealed significant increases in surface roughness parameters, such as average roughness (Ra) and root mean square roughness (Rq), in smokers. The AFM images also showed cracks and irregularities, indicating that tobacco use leads to enamel degradation, increasing susceptibility to erosion and decay. These findings align with our study, where surface roughness progressively increased with tobacco staining, highlighting a direct correlation between tobacco exposure and enamel deterioration across grades of staining.

In addition to structural damage, tobacco use significantly alters the chemical and mineral composition of enamel. Studies employing scanning electron microscopy (SEM), energy dispersive X-ray spectroscopy (EDX), and microhardness testing on smokers' and non-smokers’ enamel samples found that smoking causes demineralization and structural disorganization.^20^ Decreased microhardness and mineral content, coupled with an increased Ca/P ratio, suggest substantial enamel weakening. In our study, profilometer measurements supported these findings, showing the smoothest enamel in Grade 0 (unstained) samples, with increasing roughness across Grades 1 to 3. These results further emphasize that as tobacco staining intensifies, the enamel surface becomes rougher and more irregular, with greater amounts of residue and damage.

Tobacco use also affects the optical properties of enamel, such as its translucency and visual appearance. Previous studies have shown that cigarette smoke significantly increases enamel discoloration, while newer nicotine products like tobacco heating and vapor devices have less impact on enamel colour.^21^^,^^22^ Moreover, cigarette smoke exposure alters the chemical composition and increases the microhardness of enamel, and smoking introduces heavy metals into both enamel and dentin, exacerbating its harmful effects.^23^ In our research, photospectrometry revealed that Grade 0 enamel had the highest translucency, with greater light transmission and lower absorbance values. However, as staining increased from Grades 1 to 3, translucency significantly decreased, with lower transmission and higher absorbance, indicating reduced light penetration due to the staining. This change in optical properties underscores how smoking not only damages enamel structurally but also leads to visual alterations that negatively affect aesthetics and oral health.

Clinically, the increased surface roughness and porosity caused by tobacco use can lead to greater plaque buildup and bacterial colonization, creating the ideal conditions for dental cavities and periodontal disease.^24^ These changes also have aesthetic implications, as the browning and staining of teeth can diminish patients' self-esteem and quality of life.^25^^,^^26^ Tobacco exposure is a major risk factor for oral squamous cell carcinoma (OSCC) and other oral health conditions, including periodontitis, peri-implantitis, dental caries, and halitosis. These diseases pose significant financial and health burdens, further underscoring the need for early detection and prevention strategies.^27^^,^^28^ Secondary prevention measures, such as screening, diagnosis, and early intervention, can mitigate the progression of tobacco-related conditions. In addition, preventive services like chemoprophylaxis, screening tests, and immunizations must be continuously evaluated to address evolving public health challenges.^29^^,^^30^

While this study offers important insights into the effects of tobacco on enamel, several limitations should be noted. Potential sample selection bias may affect the generalizability of the findings, and the absence of long-term longitudinal studies limits the understanding of how tobacco-induced enamel changes progress over time. Additionally, it can be challenging to isolate the effects of tobacco from other factors, such as diet and oral hygiene practices, which may also contribute to enamel deterioration. Future research should focus on standardizing quantitative measures of enamel translucency and conducting long-term studies to explore the multifactorial influences on enamel health.^31^^,^^32^ Furthermore, developing effective clinical interventions and public health strategies aimed at reducing tobacco use will be crucial in improving oral health outcomes globally.

## Patient consent

Not applicable.

## Ethical approval

Not applicable, as the study utilized extracted teeth samples obtained from a repository, with no direct involvement of human participants.

## Ethical clearance

Ethical clearance was obtained from the Institutional Review Board.

## Funding

Nil.

## Declaration of competing interest

The authors declare that they have no known competing financial interests or personal relationships that could have appeared to influence the work reported in this paper.
